# The roles of different forms of IL-15 in human melanoma progression

**DOI:** 10.3389/fimmu.2023.1183668

**Published:** 2023-06-02

**Authors:** Sabina Di Matteo, Enrico Munari, Piera Filomena Fiore, Silvia Santopolo, Camilla Sampaoli, Andrea Pelosi, Salem Chouaib, Nicola Tumino, Paola Vacca, Francesca Romana Mariotti, Stefan Ebert, Markus Machwirth, Dorothee Haas, Marco Pezzullo, Gabriella Pietra, Melania Grottoli, Stephanie Buart, Erwan Mortier, Enrico Maggi, Lorenzo Moretta, Ignazio Caruana, Bruno Azzarone

**Affiliations:** ^1^ Tumor Immunology Unit, Bambino Gesù Children’s Hospital, Istituto di Ricerca e Cura a Carattere Scientifico (IRCCS), Rome, Italy; ^2^ Pathology Unit, Department of Molecular and Translational Medicine, University of Brescia, Brescia, Italy; ^3^ Institut national de la santé et de la recherche médicale Unitè Mixte Rechercce (INSERM UMR) 1186, Integrative Tumor Immunology and Cancer Immunotherapy, Gustave Roussy, École Pratique des Hautes Études (EPHE), Faculty De Médecine Univ. Paris-Sud, University Paris-Saclay, Villejuif, France; ^4^ Thumbay Research Institute for Precision Medicine, Gulf Medical University, Ajman, United Arab Emirates; ^5^ Immunology Research Area, Innate Lymphoid Cells Unit, Bambino Gesù Children’s Hospital, IRCCS, Rome, Italy; ^6^ Department of Pediatric Hematology, Oncology and Stem Cell Transplantation, University Hospital of Würzburg, Würzburg, Germany; ^7^ Core Facility, IRCCS Bambino Gesù Children’s Hospital, Rome, Italy; ^8^ Department of Experimental Medicine (DiMES), University of Genoa, Genoa, Italy; ^9^ Immunology Unit, IRCCS Ospedale Policlinico San Martino, Genoa, Italy; ^10^ Nantes Université, Centre national de la recherche scientifique (CNRS), Inserm, CRCI2NA, Nantes, France; ^11^ LabEx IGO, Immunotherapy, Graft, Oncology, Nantes, France

**Keywords:** cytokines, melanoma, IL-15, IL–15 complex, natural killer cells, immunology

## Abstract

**Background:**

Melanoma is a lethal skin cancer, and the risk of developing it is increased by exposure to ultraviolet (UV) radiation. The production of cytokines such as interleukin-15 (IL-15), induced by the exposure of skin cells to UV rays, could also promote melanoma development. The aim of this study is to investigate the possible role of Interleukin-15/Interleukin-15 Receptor α (IL-15/IL-15Rα) complexes in melanoma development.

**Methods:**

The expression of IL-15/IL-15Rα complexes by melanoma cells was evaluated both *ex vivo* and *in vitro* by tissue microarray, PCR, and flow cytometry. The presence of the soluble complex (sIL-15/IL-15Rα) in the plasma of metastatic melanoma patients was detected using an ELISA assay. Subsequently, we investigated the impact of natural killer (NK) cell activation after rIL-2 starvation followed by exposure to the sIL-15/IL-15Rα complex. Finally, by analyzing public datasets, we studied the correlation between IL-15 and IL-15Rα expressions and melanoma stage, NK and T-cell markers, and overall survival (OS).

**Results:**

Analysis of a melanoma tissue microarray shows a significant increase in the number of IL-15^+^ tumor cells from the benign nevi to metastatic melanoma stages. Metastatic melanoma cell lines express a phorbol-12-myristate-13-acetate (PMA)-cleavable membrane-bound IL-15 (mbIL-15), whereas cultures from primary melanomas express a PMA-resistant isoform. Further analysis revealed that 26% of metastatic patients present with consistently high plasmatic levels of sIL-15/IL-15Rα. When the recombinant soluble human IL-15/IL-15Rα complex is added to briefly starved rIL-2-expanded NK cells, these cells exhibit strongly reduced proliferation and levels of cytotoxic activity against K-562 and NALM-18 target cells. The analysis of public gene expression datasets revealed that high IL-15 and IL-15Rα intra-tumoral production correlates with the high levels of expression of CD5^+^ and NKp46^+^ (T and NK markers) and significantly correlates with a better OS in stages II and III, but not in stage IV.

**Conclusions:**

Membrane-bound and secreted IL-15/IL-15Rα complexes are continuously present during progression in melanoma. It is notable that, although IL-15/IL-15Rα initially promoted the production of cytotoxic T and NK cells, at stage IV promotion of the development of anergic and dysfunctional cytotoxic NK cells was observed. In a subgroup of melanoma metastatic patients, the continuous secretion of high amounts of the soluble complex could represent a novel NK cell immune escape mechanism.

## Introduction

Cutaneous melanoma is the most lethal form of skin cancer both in adult and pediatric patients and is able to metastasize during the early stages of tumor development. Its prevalence increases at a rate of about 3% annually (1), representing a significant public health problem. An estimated 160,000 new cases per year are diagnosed worldwide. The incidence of melanoma increases as a function of age, rising from 1.1 per million in 1- to 4-year-olds to 10.4 per million in 15- to 19-year-olds. In addition, starting from the 1970s, an increase in the number of pediatric melanomas was observed, with a mean percent per year ranging between 2% and 2.9% (1–3). Different parameters contribute to determining the staging of melanoma such as tumor thickness, ulceration, mitotic rate, and metastases formation into local lymph nodes and distal sites. Most early-stage melanomas (i.e., those at stage I or II) are classified as non-metastatic, even though ~ 5% of affected patients will experience recurrence. In contrast, stage III melanomas are characterized by local metastases, and in stage IV the presence of distant metastasis is typical (1–3). It has been shown that exposure to ultraviolet (UV) radiation increases the risk of developing melanoma (3). In recent years, several studies have demonstrated how UV exposure can induce inflammation and the production of different cytokines (4, 5). In this study, we hypothesize that cytokines could be somehow involved in melanoma development. In this context, Mohamadzadeh et al. found that UV-B rays upregulate the expression of interleukin-15 (IL-15) in human skin cells (6). Therefore, we focused on this cytokine.

IL-15 is a pleiotropic cytokine, linking *in vivo* innate and adaptive immune responses (7). It is included into the four α-helix bundle cytokine family and is produced constitutively by both normal and neoplastic cells (8). IL-15 stimulates immune cell responses through an intermediate-affinity heterodimeric receptor (7) shared with IL-2 (IL-2Rβ/γ_c_). This may or may not be associated with the high-affinity private IL-15Rα chain, which confers specificity and faciliatates signal transduction (9, 10). Moreover, specific IL-15Rα isoforms associating intracellularly with IL-15 (11) are subsequently expressed at the cell surface as a membrane-bound IL-15 complex (mbIL-15). This complex may signal in *cis* (12), but essentially transmits the signal in *trans* to neighboring cells equipped with the intermediate affinity receptor (11, 13, 14). The existence of a transmembrane-bound form of IL-15 (not bound to IL-15Rα) that is resistant to acidic shock has also been reported; this form not only is able to signal in *trans* but can also activate a reverse signal upon stimulation with soluble IL-15 receptor-alpha or anti-IL-15 antibodies (15). Both mb-IL15 and transmembrane mb-IL-15 behave as hyperagonist isoforms. In addition, we reported that the IL–15/IL-15Rα heterodimer is also detectable as a soluble complex (sIL-15/IL-15Rα complex) that interacts with cells expressing the intermediate affinity IL-15R. This complex behaves as a hyperagonist isoform, displaying greater half-life, bioavailability, and biological efficacy than monomeric IL-15 (13, 16). In this context, Muller et al. have identified the IL-15Rα isoforms (WT IL-15Rα and IC3 IL-15Rα) that are specifically involved in the assembly of different IL-15/IL-15Rα complexes (11).

The administration of exogenous IL-15 is of major therapeutic interest in immuno-oncology (7, 17), promoting the expansion of both natural killer (NK) and T cells (17, 18). In NK and T cells, IL-15, more than other cytokines, leads to enhanced anti-tumor immunity (18, 19). Indeed, recombinant human IL-15 and sIL-15/IL-15Rα, employed alone or in association with other therapies, cause efficient tumor regession in several experimental tumor models (18, 19).

Although IL-15 is a promising therapeutic tool, several human solid tumor-derived cell lines and tissues, such as from the colon, head and neck, kidney, lung, triple-negative breast cancers, and melanoma, including uveal melanomas, express intra-tumoral IL-15 and/or IL-15Rα, have been shown to act as tumor promoters and, in a few instances, as protecting factors (8). Concerning human melanomas, it is known that long-term survival correlates with low levels of monomeric IL-15 in the serum, whereas a poor survival outcome correlates with high levels of monomeric IL-15, possibly reflecting the increased expressions of T cell immunoglobulin and mucin-domain containing-3 (TIM-3) and programmed cell death protein 1 (PD-1) in both NK and T cells (20). Barzegar et al. showed that IL-15 produced by primary cutaneous melanoma cells, acting through “juxtacrine mechanisms”, triggers the activation of the pro-inflammatory and immune-regulatory NF-κB pathway and the modulation of class I major histocompatibility complex (MHC) molecule expression, which favors immune-escape mechanisms from cytolytic T cells (21). In addition, a study on human uveal melanoma shows that IL-15 administration induces the proliferation of all tested cell lines and decreases their susceptibility to NK cell-mediated cytotoxicity and the response to cisplatin treatment (22). Moreover, Bergamaschi et al. detected high levels of sIL-15/IL-15Rα in the sera of eight lympho-depleted metastatic melanoma patients (23). In addition, several preclinical studies have reported that prolonged *in vivo* stimulation with sIL-15/IL-15Rα complexes leads to the accumulation of exhausted NK cells (24–27). However, the impact of sIL-15/IL-15Rα complexes on melanoma progression, and their roles in the development of an immunosuppressive tumor microenvironment (TME) and the establishment of NK and T-cell exhaustion, have yet to be clarified.

In this context, our study aims to investigate if (i) IL-15 expression correlates with tumor stage development (by analyzing melanoma tissue microarrays), (ii) melanoma cells are competent for the assembly and production of IL-15/IL-15Rα complexes, (iii) the presence of elevated plasmatic levels of the sIL-15/IL-15Rα complex in metastatic melanoma patients depends on lympho-depletion, (iv) prolonged *in vitro* exposure of NK cells to the sIL-15/IL-15Rα causes direct dysfunctions in these cells and (v) IL-15/IL-15Rα expression correlates with tumor progression, as revealed by the analysis of public datasets in melanoma patients.

## Material and methods

### Cell lines and treatments

The metastatic human melanoma cell lines MeWo, C32, A2058, SK-Mel-28, SH-4, A375, Hs 852.T, the human erythroleukemia cell line K562, the B cell leukemia NALM-18, and the primary/metastatic pair WM-115/WM-266–4 (derived from the same patient) were purchased from the American Type Culture Collection (ATCC, Virginia, USA). The primary T1 and the metastatic melanoma G1 cell lines, derived from the same patient, were obtained from the Institute Gustave Roussy (Villejuif, France). The abovementioned cell lines were expanded in RPMI-1640 medium (Euroclone, Italy) supplemented with 2 mM L-glutamine (Euroclone), 1% streptomycin and penicillin (Euroclone), and 10% fetal bovine serum (FBS; Thermo-Fisher Scientific, Massachusetts, USA). In some experiments, melanoma cell lines were treated for 36 h with 1 ng/mL of interferon alpha (IFN-α) (Merck, Canada) and 1 ng/ml of anti-CD40 monoclonal antibodies (mAb) (clone 5C3 - Functional Grade, Thermo-Fisher Scientific). In other experiments, WM-115, WM-266–4, T1, G1, A2058, and SK-MEL-28 melanoma cells were treated with 100 ng/mL of phorbol-12-myristate-13-acetate (PMA, Sigma-Aldrich) for 3 h as previously described (28). All cell lines were confirmed as mycoplasma-negative by reverse transcription PCR (RT-PCR) (Eurofins, Luxembourg).

### Purification of NK cells and cytokine treatments

Freshly isolated NK cells were purified from the fractions of peripheral blood mononuclear cells (PBMC) derived from healthy donors’ buffy coats using the RosetteSep Human NK cell Enrichment Cocktail (Stemcell Technologies, Canada) in accordance with the manufacturer’s instructions. NK cells were cultured as previously described (29).

Freshly isolated NK cells were treated for 5 days with IL-15clpx (16) or monomeric recombinant human IL-15 (rIL-15) at different concentrations. IL-15clpx is a fusion protein consisting of human IL-15 and human IL-15Rα-sushi linked by a flexible peptide, this complex behaves as a potent superagonist of IL-15Rβ/γ. Indeed, IL-15clpx induces the proliferation of the MO7 IL-15Rβ^+^/γ^+^ cell line, with EC50s ∼ 25 pM far lower than that of rIL-15 alone 3 nM (16).

Alternatively, freshly isolated NK cells were expanded over 12 days in RPMI-1640 medium (Euroclone) supplemented either with 10% FBS and 600 U/mL recombinant human IL-2 (rIL-2 Proleukin; NovartisFarma, Italy) or with rIL-15 at 20 ng/mL (Miltenyi Biotec, Italy). Thereafter, a fraction of NK cells were starved for 36 h and then re-fed for an additional 5 days with rIL-2 at 600 U/mL (starved-Ctrl) or rIL-15 at 20 ng/mL or IL-15clpx at 1 ng/mL or 0.5 ng/mL (29). After starvation, NK cells we also re-fed, in the abovementioned conditions, with medium changes every 3 days. Finally, activated NK cells were used as effector cells against CellTracker Green labeled K562 cells employed as targets at different effector : target (E:T) ratios.

### Cytotoxicity assays

NK cytotoxicity activity was evaluated using K562 and NALM-18 cells as targets. Tumor cells were stained with 5 μM Cell Tracker Green (CMFDA; Thermo-Fisher Scientific) and incubated with NK cells at 37°C at different E:T ratios. Four hours later, tumor cell suspensions were exposed to propidium iodide (PI, Sigma-Aldrich) at a 1:200 ratio, and cell viability was determined using flow cytometry (Cytoflex S Beckman-Coulter). Dead target cells were recognized as CMFDA^+^ PI^+^ and the percentage (%) of cell lysis was calculated as reported before (30).

### Immunohistochemistry studies

A melanoma tissue microarray with duplicate cores per case (ME1004h, D.B.A. Italia s.r.l.), including both pediatric and adult samples, was deparaffinized and unmasking of the antigenic epitopes was performed using PT link (DAKO, Italy). Incubation with 3% H_2_O_2_ solution in methanol was employed to block endogenous peroxidase activity, and treatment with 5% bovine serum albumin (BSA) and 1% goat serum in phosphate buffer saline (PBS) blocked non-specific sites. Slides were incubated overnight at room temperature with anti-IL-15 monoclonal antibodies (mAb) (Abcam) in a humidified chamber. A Dako EnVision FLEX Mini Kit (Dako) was employed for detection, followed by counterstaining with Gill’s hematoxylin. Subsequently, the slides were scanned with a NanoZoomer S60-Digital slide scanner (Hamamatsu-Photonics, Hamamatsu, Japan). Finally, the images were processed using NDP.view2 software (Hamamatsu-Photonics). Slides were evaluated independently by pathologists in a blinded fashion.

### ELISA assay

Plasma from non-lympho-depleted metastatic melanoma patients was collected at different time intervals, ranging between 2 and 6 months (T1 and T2), divided into 500-μL aliquots, and rapidly frozen at –80 C.

Subsequently, aliquots of each sample were thawed, and quantification of sIL-15/IL-15Rα complex was performed by ELISA assay human IL-15/IL-15Rα complex DuoSet ELISA (R&D Systems) in accordance with the manufacturer’s instructions. ELISA assays performed at different time intervals on plasma aliquots derived from the same patients yielded homologous results, confirming the stability of the sIL–15/IL-15Rα complexes over time under cryostorage conditions, as previously stated (23).

### Polymerase chain reaction

Total RNA was prepared using an RNeasy mini kit in accordance with the manufacturer’s instructions (Qiagen) from primary and metastatic human melanoma cell lines stimulated or not with an interferon-α/anti-CD40 monoclonal antibodies (mAb) combined treatment (IFN-α/anti-CD40) or PMA. Subsequently, transcription into cDNA of 1 μg of RNA was performed using an oligo-dT primer with high-capacity cDNA reverse transcription kit (Thermo Fisher Scientific).

All PCR amplifications were performed with the following parameters: 3 min at 94°C, followed by 35 cycles of 95°C for 15 s, 60°C for 15 s, and 72°C for 90 s, with a final extension at 72°C for 5 min. The amplification of the entire coding region of human IL-15Rα required a nested step, in which 1 μL of the first PCR was used as a template for the nested step. 18S rRNA was used as an internal control. The primers used for PCR are IL-15Rα first round FW: 5′-GTGTCCTGTGGAGCTGCCGCCATG-3’, RV: 5′-CCCGCTTCCTTGCACCTCTTCTCAGTCGTC-3’; IL-15Rα second round FW: 5′-GGCGCTGCTACTGCTGCTGCTGCTCC-3’, RV: 5′-GGGAGATGAAGCTGCGGGCTCTTTTC-3’. IC3 FW: RV: 5′-CGTGCCGGCTTTACGCTTGA-3’; 18S FW: 5′- GGAGAGGGAGCCTGAGAAA -3’, RV: 5′- CGAAAGAGTCCTGTATTGTTATTT -3’; ADAM 17 FW 5′-ACTGCACGTTGAAGGAAGGT-3’, RV 5′ -ACGCCTTTGCAAGTAGCATT - 3’, GAPDH FW 5′-TCTTTTGCGTCGCCAGCCGA-3’, RV 5′-ACCAGGCGCCCAATACGACC -3’.

### Flow cytometry

For the detection of mbIL-15, melanoma cells were stained with the anti-human IL-15 monoclonal AF700-conjugated antibody monoclonal antibodies (mAb) (IC2471N; R&D System, Minnesota USA) for 20 min at 4°C. Cells were analyzed using the Beckman-Coulter Cytoflex-S flow cytometer (Beckman-Coulter, California, USA). At least 10,000 events for each condition were analyzed. The acquired data were analyzed using CytExpert-2.3 (Beckman-Coulter) and FlowJo v.10 software (BD-Biosciences, New Jersey, USA).

### Statistical analysis

Data were expressed as mean ± standard deviation (SD). A paired Student’s two-tailed *t*-test or two-way analysis of variance (ANOVA) associated with a Bonferroni correction in case of multiple comparisons was employed for the calculation of the statistical significance, as indicated. *P*-values < 0.05 were considered statistically significant.

Graphs were generated using GraphPad Prism 6 software (GraphPad Software, Boston, MA, USA). Overall survival (OS) probabilities were estimated using Kaplan–Meier survival curves with a log-rank test associated with Bonferroni correction. We were able to measure differences between stages using tools present in R2 and Biomedical Informatics Institute datasets (http://r2.amc.nl, https://bioinfo.henu.edu.cn). The last quartile of the distribution was used as the cut-off to split the samples into high and low antigen expression groups.

## Results

### IL-15 is expressed *ex vivo* in human melanoma cells

**Figure 1:**
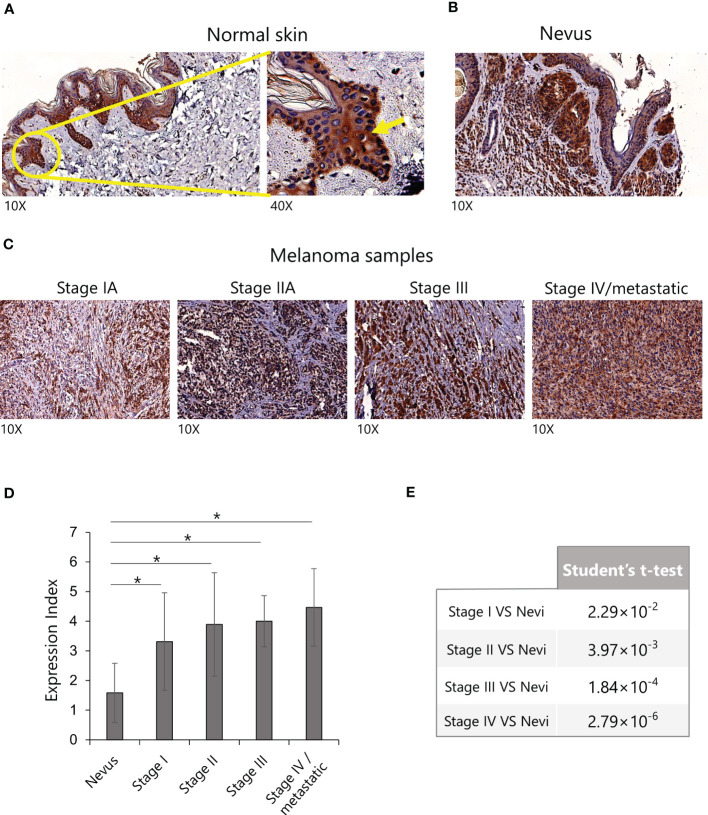
Expression of IL-15 in human melanoma tissue microarrays by immunohistochemistry. **(A)** Representative images of sections of normal skin, **(B)** nevi, and **(C)** melanomas (from stage I to stage IV) stained with anti-human IL-15 monoclonal antibodies (mAb). **(D)** Expression index of IL-15 staining in the indicated IHC. p-values were calculated using a Student’s t-test (*p < 0.05). **(E)** Results of Student’s t-test relative to graph in **(D)**. IHC, immunohistochemistry; IL-15, interleukin-15.

As a first step, we investigated the expression of IL-15 in samples of normal skin, nevi, and melanomas at different stages using a tissue microarray. In normal skin, IL-15 was mainly detected in the epidermis (**Figure 1A**) in the basal cell layer (**Figure 1A** yellow arrow). In contrast, in benign nevi (**Figure 1B**), well-delimited nests of melanocytes positive for IL-15 were detected in the dermis, whereas only a faint signal was detected in the epidermis. In stage I, II, and III melanoma samples (**Figure 1C**), a higher level of expression and distribution of IL-15^+^ tumor cells were detected in the dermis and epidermis (*p* < 0.05) than in the nevi, whereas in metastatic samples virtually all tumor cells were strongly positive (*p* < 0.05 *vs*. nevi). Furthermore, statistical analysis revealed a significant difference (*p* < 0.05) in the expression intensity of IL-15 in melanoma samples compared with nevi (**Figures 1D, E**). We also found an increase in the expression of IL-15-positive melanocytes among stages, but this increase was not statistically significant.

### Definition of IL-15Rα transcript isoforms involved in the assembly of IL-15/IL-15Rα complexes in primary and metastatic melanoma cell lines

**Figure 2:**
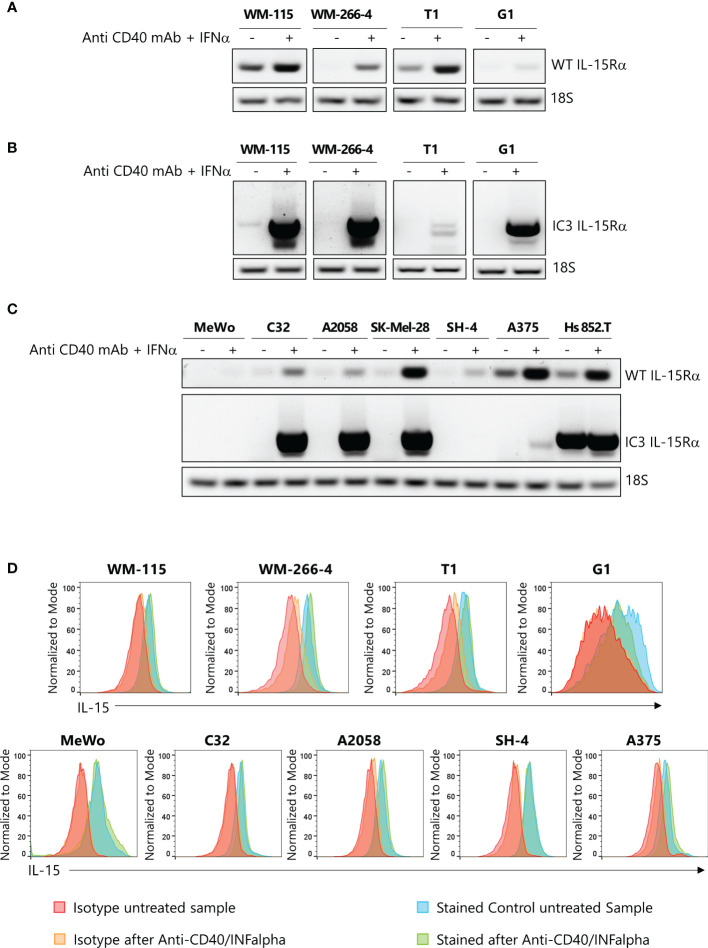
Expression of IL-15Rα isoforms and of mbIL-15 in primary and metastatic melanoma cell lines. Relative expression by PCR of Wild Type (WT) IL-15Rα **(A)** and of IC3 IL-15Rα **(B)** transcripts in two pairs of primary and metastatic melanoma cell cultures derived from the same patients, stimulated or not with anti-CD40/IFN-α. 18s RNA expression was used as loading control **(C)** Relative expression of WT IL-15Rα and of IC3 IL-15Rα mRNA transcripts in seven other melanoma cell lines, stimulated or not with anti-CD40/IFN-α. 18s RNA expression was used as loading control **(D)** Flow cytometry analysis of the surface expression of mbIL-15 in two pairs of primary and metastatic melanoma cell lines and other melanoma tumors stimulated, or not stimulated, with anti-CD40/IFN-α. IFN-α, interferon alpha; mbIL-15, membrane-bound IL-15 complex.

Subsequently, several melanoma cell lines were analyzed by PCR for the expression of IL-15Rα transcripts, previously reported by Muller et al. (11), to be involved in the assembly of the mbIL-15 isoform, WT IL-15Rα, and the IL-15/IL-15Rα soluble complex (IC3 IL-15Rα). First, we analyzed two pairs of primary/metastatic melanoma cell lines (WM-115/WM-266–4 and T1/G1), each derived from the same patient, and seven other metastatic melanoma cell lines. The assay was performed before and after stimulation with IFN-α and anti-CD40 monoclonal antibodies (mAb), a treatment that, *in vivo*, induces the secretion of the IL-15/IL-15Rα soluble complex (31). First, we focused our attention on WT IL-15Rα and IC3 IL-15Rα expression on primary/metastatic pairs of tumors. PCR analysis revealed that only the primary samples (WM-115 and T1) constitutively expressed the WT IL-15Rα transcript, and this expression was strongly upregulated upon IFN-α/anti-CD40 stimulation (**Figure 2A**). Concerning the IC3 IL-15Rα transcript, we observed an increase in its expression following stimulation with IFN-α/anti-CD40 monoclonal antibodies (mAb) (**Figure 2B**).

Second, we analyzed the expression of the two IL-15Rα isoforms on the remaining melanoma cell lines. We observed that the WT IL-15Rα transcript was constitutively expressed in five/seven lines (weakly in three of them). However, this transcript was strongly upregulated after IFN-α/anti-CD40 monoclonal antibodies (mAb) stimulation in all the cell lines, except in the MeWo cell line (**Figure 2C**, upper panel). In addition, we found that only Hs 852.T cell line constitutively expressed the IC3 IL-15Rα transcript. We also found that, in this case, the IFN-α/anti-CD40 monoclonal antibodies (mAb) treatment induced the expression of this isoform on five/seven tested cell lines (**Figure 2C**, lower panel).

Last, we used flow cytometric analysis to reveal the expression of the mbIL-15 isoform in both primary/metastatic pairs of tumors and in the seven melanoma cell lines. **Figure 2D** shows that, in all cases, tumor cells constitutively expressed the mbIL-15 whose expression was not modified after IFN-α/anti-CD40 monoclonal antibodies (mAb) treatment. Interestingly, the MeWo cell line which lacked both WT IL-15Rα and IC3 IL-15Rα transcripts expressed mbIL-15. This isoform could likely represent the so-called trans-membrane IL-15 isoform that is not retained at the cell surface by any IL-15Rα isoform (15).

To further determine which isoform was predominant, we performed an ELISA to detect the presence of soluble complex. No soluble complex was detected in any of the samples analyzed, even after lipopolysaccharides (LPS) cell treatment (data not shown) (31). This could be due to the presence, in the IC3 IL-15Rα isoform, of a retention sequence of the endoplasmic reticulum that limits its availability (11). It is possible that additional co-stimuli are necessary *in vitro* to trigger the secretion of the sIL-15/IL-15Rα complex. These data clearly indicate that mbIL-15 is the dominant isoform in melanoma cells.

### Modulation of the surface expression of the mbIL-15 by PMA

mbIL-15/IL-15Rα complexes are cleaved into biologically active soluble molecules by disintegrin and metalloproteinase-17 (ADAM17) (31) that is, in turn, activated by IL-15, limiting the proliferation of NK cells (32). Using these data, we determined that the mbIL–15/IL-15Ra complex expressed by melanoma cells could be cleaved by ADAM17, and thus we treated the melanoma cells with PMA, a powerful activator of ADAM17 (33).

**Figure 3:**
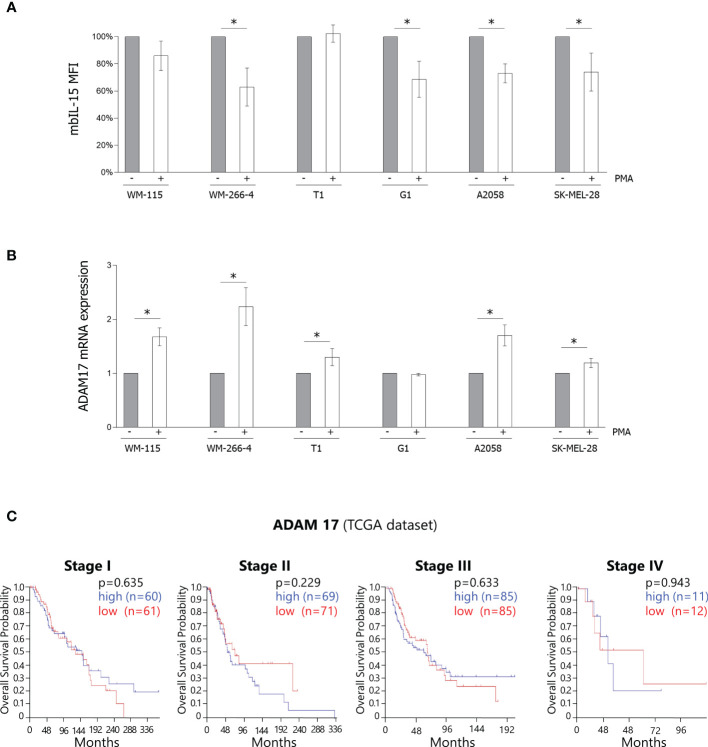
Modulation of the surface expression of the mbIL-15 by PMA. **(A)** Surface expression of mbIL-15 before and after treatment with PMA at 100 ng/mL for 3 h was measured by flow cytometry on primary/metastatic pairs each derived from a single patient (WM-115/WM-266–4 and T1/G1) or on metastatic cell lines (A2058 and SK-MEL-28). Bar graphs show the MFI expressed as a percentage of the untreated controls. The results are the means ± SD (*n* = 3); *p*-values were calculated using a Student’s t-test (*p < 0.05). mbIL-15, membrane-bound IL-15 complex; MFI, mean fluorescence intensity; PMA, phorbol-12-myristate-13-acetate. **(B)** RT-PCR analysis of ADAM17 mRNA was performed on the abovementioned samples before and after treatment with PMA. ADAM17 mRNA levels were normalized with GAPDH and were expressed as fold increases relative to the untreated controls. The results are the means ± SD (*n* = 3); *p*-values were calculated using a Student’s t-test (*p < 0.05). ADAM17, disintegrin and metalloproteinase-17; GAPDH, glyceraldehyde 3-phosphate dehydrogenase; PMA, phorbol-12-myristate-13-acetate; RT-PCR, reverse transcription PCR. **(C)** Analysis of the public dataset TCGA concerning the correlation between ADAM17 expression and OS in melanoma stages I–IV. ADAM17, disintegrin and metalloproteinase-17; OS, overall survival; TCGA, The Cancer Genome Atlas.

Flow cytometric analysis (**Figure 3A**) shows that, in both primary/metastatic pairs of melanoma cultures, PMA treatment (100 ng/mL for 3 h) causes a statistically significant decreased surface expression of mbIL-15/IL-15Rα (*p* < 0.05) in the metastatic samples (WM-266–4, G1, A2058, and SK-MEL28) but not in the primary ones (WM-115 and T1). Interestingly, our data show that PMA treatment causes, in all the cell lines analyzed, except in G1 cells, significant increased ADAM17 RNA levels (**Figure 3B**). Therefore, due to the impact of ADAM17 on the cleavage of mIL-15/IL-15Rα complexes, we queried the public data set TCGA for the correlation of ADAM17 expression and OS, observing no correlation even within the stages (**Figure 3C**).

### High levels of circulating sIL-15/IL-15Rα complexes are present in a fraction of metastatic melanoma patients

**Figure 4:**
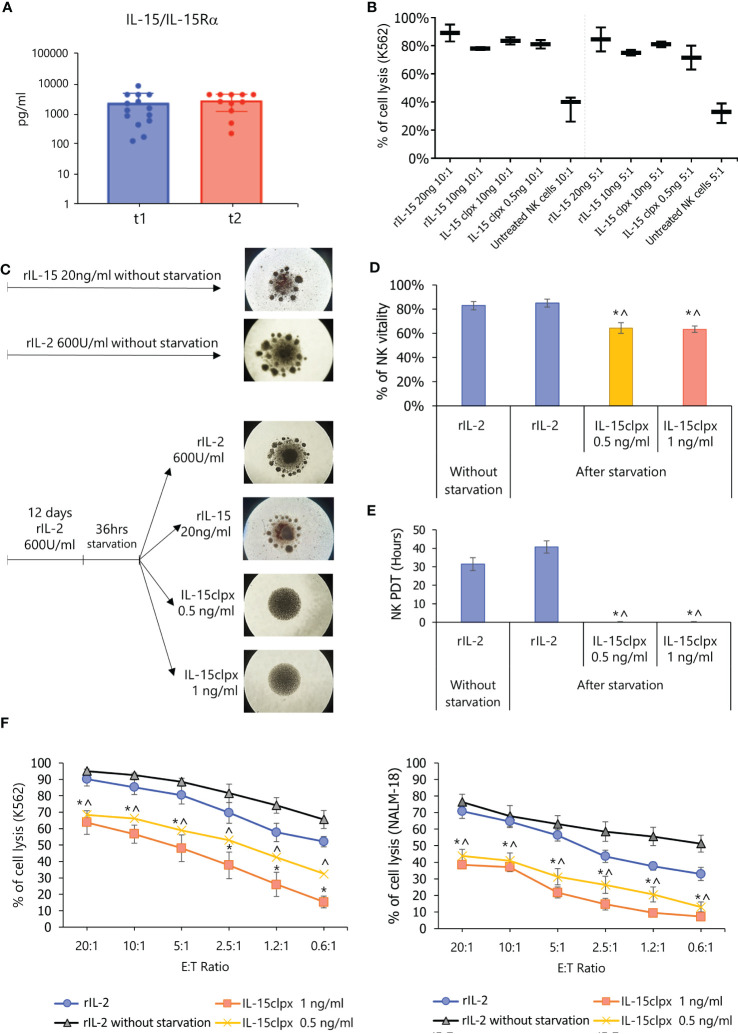
Detection of sIL-15/IL-15Rα complex in plasma from metastatic melanoma patients and effect of long-lasting stimulation of NK cells with IL-15clpx. **(A)** Detection of the sIL-15/IL-15Rα complex in the plasma of non-lympho-depleted metastatic melanoma patients. Dot-Plot analysis of the levels of sIL-15/IL-15Rα complex (presented on a log scale) in the plasma from 62 metastatic melanoma patients at two different time points with intervals ranging between 2 and 6 months. **(B)** NK cytotoxicity assays against 5-Chloromethylfluorescein diacetate (CMFDA)-labeled K562 target cells at ratios of 10:1 and 5:1 E:T. Data were expressed as mean ± SD (*n* = 3) of the percentage of cell lysis (PI^+^ cells). Freshly isolated NK cells were stimulated for 5 days with different concentrations of rIL-15 (20 and 10 ng/mL) and of the IL-15clpx (10 ng/mL and 0.5 ng/mL). Non-stimulated NK cells were used as controls (untreated NK cells). The two-way ANOVA with Bonferroni’s multiple comparisons test was used for the calculation of the statistical significance among the different cohorts. No significant differences were observed within various dose ratios. A significant difference (*p* < 0.05) was found only between the different dose/ratio of rIL-15 or IL-15clpx and the untreated control. ANOVA, analysis of variance; NK, natural killer; **(C)** Schematic schema of long-term stimulation of NK cells. On the right, representative images of the NK cells at the end of the long-term stimulation protocols have been reported. It is possible to observe the formation of clusters in the control wells treated with rIL-2 and rIL-15, which, however, are not present in the wells treated with IL-15clpx. The images are representative of *n* = 5 experiments, × 4 magnification. NK, natural killer. **(D)** Analysis of the NK viability after long-term stimulation. Data were expressed as mean ± SD (*n* = 3) of the percent of NK^PI-^ cells. *p < 0.05 vs rIL-2 without starvation. ^ *p* < 0.05 vs. rIL-2. **(E)** Analysis of the proliferation (PDT, population doubling time) of long-term stimulated NK cells. Data were expressed in the mean of hours ± SD (*n* = 3) of percent of cell lysis (PI^+^ cells). *p < 0.05 vs. rIL-2 without starvation. ^ *p* < 0.05 vs rIL-2. NK, natural killer. **(F)** Analysis of the long-term IL-15clpx or rIL-2 stimulation on the cytotoxic potential of activated NK cells. Allogeneic activated NK cells were used as effector cells against Cell Tracker Green-labeled K562 and NALM-18 cells used as targets at different E:T ratios. Data were expressed as mean ± SD (*n* = 3) of percent of cell lysis (PI^+^ cells). *p < 0.05 vs rIL-2 without starvation. ^ *p* < 0.05 vs rIL-2. E:T, effector:target; NK, natural killer.

A previous study reported that plasma samples from lympho-depleted metastatic melanoma patients were characterized by high levels of the sIL-15/IL-15Rα complex (23). However, no data have been reported in non-lympho-depleted patient population. Therefore, we analyzed, at two different time points (T1 and T2), the presence of plasmatic sIL-15/IL-15Rα complex in 62 non-lympho-depleted metastatic melanoma patients. A threshold of 100 pg/mL was established for defining concentrations that were biologically significant. We found that most of the samples exhibited sIL-15/IL-15Rα complex levels below this threshold. However, a fraction of patients (14 out of 62) displayed high plasmatic levels of the soluble complex at T1, and 11 of these patients (78%) maintained the same levels of the soluble complex at T2 of analysis (**Figure 4A**).

### The prolonged *in vitro* exposure to IL-15 hyperagonist inhibits NK cell cytotoxicity

Based on the abovementioned data, we hypothesized that a prolonged *in vivo* exposure of effector NK cells to sIL-15/IL-15Rα may lead to the exhaustion of these cells (26, 27). To prove this hypothesis, we evaluated the functional impact of sIL-15/IL-15Rα on NK cells *in vitro*. **Figure 4B** shows that 0.5 ng/mL IL-15clpx (17) administered at day 5 could induce cytolytic activity in freshly isolated NK cells against K562 target cells with an efficacy similar to that obtained by 20 and 10 ng/mL of IL-15 (i.e., doses routinely employed for NK cell stimulation) (27). Freshly isolated untreated NK cells were employed as controls and exhibited a significant reduction in cytolytic activity against K562 cells in comparison with the stimulated cohorts, but we did not observe any significant difference in cytolytic activity among the stimulated cohorts. Thus, we administered IL-15clpx at 1 and 0.5 ng/mL for the forthcoming experiments in which freshly isolated NK cells were first cultured for 12 days in the presence of rIL-2 at 600 UI/mL or of rIL-15 at 20 ng/mL. In **Figure 4C**, cluster formation indicated that NK cells had proliferated. Thereafter, part of the NK cells was starved for 36 h (i.e., with no rIL-2) and subsequently re-fed with rIL-2 or rIL-15 at 20 ng/mL, or IL-15clpx (at 0.5 or 1 ng/mL) for an additional 5 days (**Figure 4C**). After starvation, NK cells re-fed with rIL-2 or recombinant IL15 still displayed cluster formation (**Figure 4C**) and exhibited cell viability and growth rates similar to those of the control group, which was continuously supplemented with rIL-2 (**Figures 4D, E**). These cells also displayed high levels of cytolytic activity against K562 and NALM-18 target cells (**Figure 4F**). By contrast, NK cells re-fed with IL-15clpx exhibited reduced proliferative activity, as shown by the inhibition of aggregates formation (**Figure 4C**) and the almost total inhibition of the population doubling time (PDT), without significant loss of cell viability (**Figures 4D, E**). In addition, these NK cells also exhibited low levels of cytotoxic activity against K562 and NALM-18 cells in comparison with NK cells re-fed with rIL-2 (**Figure 4F**) or rIL-15 (data not shown).

### High IL-15 and IL-15Rα expression correlates with a favorable OS in stages II and III but not in stage IV

To confirm and extend our *in vitro* results, we queried public gene-expression datasets for the impact of intra-tumoral IL-15 and IL-15Rα expression on the overall patient’s survival.

**Figure 5:**
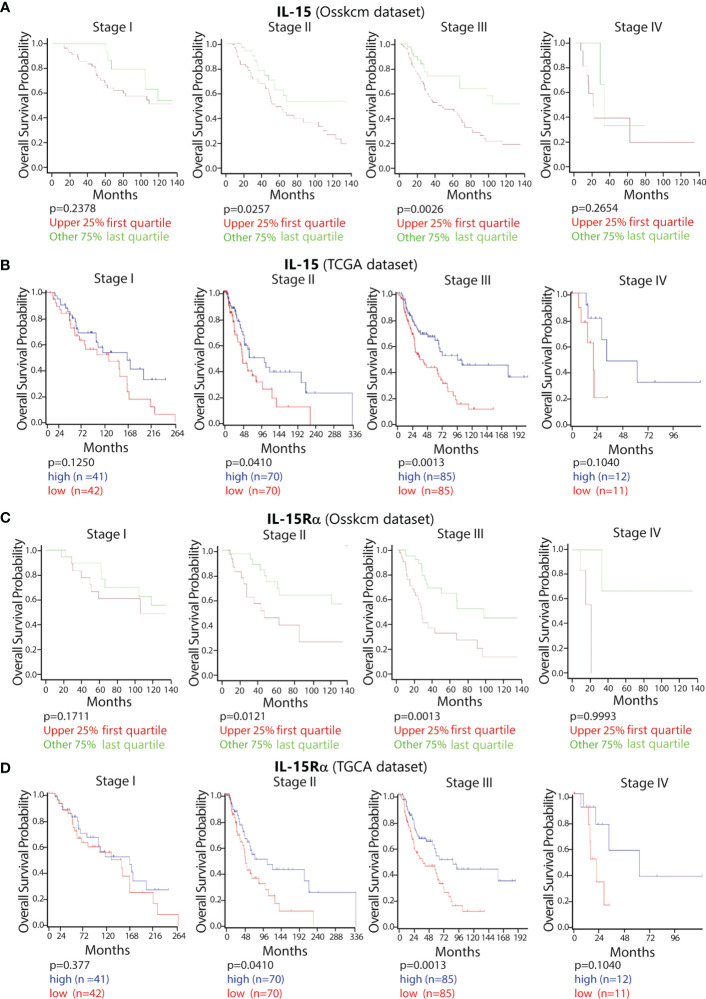
Impact of the high expression of IL-15 and IL-15Rα on the OS in melanoma patients at the different stages of disease. Using the public datasets (OSskcm and TCGA) of patients with melanoma, the OS probabilities were calculated according to the level of expression of intra-tumoral IL-15 (**A** OSskcm and **B** TCGA) and IL-15Rα (**C** OSskcm and **D** TCGA) from stage I to stage IV. The samples were divided into categories of high vs. low antigen expression using the last quartile of their distribution as a cut-off value. IL-15, interleukin-15; OS, overall survival; TCGA, The Cancer Genome Atlas.

Thus, two public datasets, including both adult and pediatric patients, were analyzed, namely, OSskcm (1,085 patients) and TCGA (470 patients). Analysis of the correlation among different disease stages, expression of IL-15, and patient’s OS was performed. In stages II and III, a higher level of expression of IL-15 was significantly correlated with a better clinical outcome (OSskcm. *p* = 0.026/0.041 and TCGA 0.003/0.001, respectively), while this correlation was not found in stages I and IV (**Figures 5A, B**). In order to confirm these data, a third dataset including only metastatic patients (Bhardwaj) was used. In addition, we found a similar correlation between the level of IL-15 and OS (data not shown). The same analysis was also conducted for IL-15Rα, revealing that high levels of IL-15Rα correlated with a better OS in patients at stages II and III, but levels of expression not at stages I and IV in both the OSskcm and TCGA datasets (**Figures 5C, D**). This combined dataset analysis involving IL-15 and IL-15Rα strongly suggests that, since the two molecules follow a very similar trend in the two datasets, they could act *in vivo* as hyperagonist membranes or soluble complexes.

### High levels of CD5 and NKp46 expression positively correlate with the level of expression of IL-15 and IL-15Rα

Based on these data, we then analyzed the correlation between of IL-15 and IL- 15Rα and the expression of CD5, a marker present on T cells (34), and the expression of NKp46 an activating receptor virtually specific for NK cells (35). As shown in **Supplemental Figure 1A**, a strong positive correlation existed between the expression of IL-15, IL-15Rα, and CD5 (*p* = 4.53·10–^35^ and *p* = 1.3·10–^115^, respectively) and NKp46 (*p* = 5.22·10–^24^ and *p* = 4.35·10–^35^, respectively).

### High levels of CD5 and NKp46 expression positively correlate with OS in stages II and III but not in stage IV

Within the same dataset, we analyzed the correlation between CD5 (**Supplemental Figure 1B**) and NKp46 (**Supplemental Figure 1C**) expression and OS at different stages of the disease. We observed that in stages II and III, high levels of CD5 or NKp46 expression translated to a better OS (*p* = 6.2 ·10–^3^ and *p* = 0.034 for CD5, and *p* = 0.026 and *p* = 0.032 for NKp46), but this was not the case in stage IV.

## Discussion

Since in melanoma patients’ high monomeric IL-15 serum levels have been reported to correlate with a poor clinical outcome (20), we asked what could be the consequences of a too-high and constant production of the IL-15/IL-15Rα complex.

Many studies have demonstrated that this molecule exerts very powerful biological effects on NK cells. In one of these studies, NK cells, expanded with the sIL-15/IL-15Rα complex *in vivo*, mediated prompt skin allograft rejection in the absence of any adaptive immune cell involvement (36). Importantly, other studies also suggested that the presence of the IL-15/IL-15Rα complex within the TME may represent a double-edged sword. Indeed, in pre-clinical melanoma models, the presence in melanoma cells and/or stromal cells of the IL-15/IL-15Rα complex (either in membrane-bound or soluble form) (37, 38), or treatment with IL-15clpx, may lead to the inhibition of tumor progression and reduction of the lung and liver metastases (39). On the other hand, prolonged *in vivo* stimulation with sIL-15/IL-15Rα has been shown to cause NK cell exhaustion (25, 26, 40). Since NK cells play a major in the control of the metastatic spread (41), we hypothesized that the detection of high levels of sIL-15/IL-15Rα in the plasma of both lympho-depleted and non-lympho-depleted metastatic melanoma patients (24) could contribute to the exhaustion of NK cells in these patients.

In this study, we confirmed this hypothesis by investigating if (i) normal and neoplastic melanocytes express IL-15 by the analysis of a melanoma tissue microarray, (ii) melanoma cell lines produce the IL-15/IL-15Rα complexes either spontaneously or under inflammatory stimuli, (iii) the plasma from non-lympho-depleted metastatic melanoma patients is a major source of the sIL-15/IL-15Rα, (iv) the exhaustion of NK cells by the IL-15clpx is an *in vivo* phenomenon linked to particular cell interactions (26) or, alternatively, could be due to direct activity on NK cells and therefore reproducible *in vitro* as proposed elsewhere (27), and (v) IL-15 and IL-15Rα expression correlates with tumor progression, by analyzing a public dataset on melanoma patients.

We analyzed a tissue microarray to find possible correlations between intra-cellular expression of IL-15 and tumor stage and evolution. In the first set of experiments, we found that IL-15 is detectable in the basal layer of normal epidermis. Notably, the skin plays a fundamental immune-neuro-endocrine function through the production, by epidermic keratinocytes and melanocytes, of several immunomodulatory cytokines and chemokines (42, 43). Thus, it is conceivable that IL-15 could play a physiologic role in skin-related immune responses (6), fostering, for instance, cutaneous tissue-resident memory T cells (T_RM_) (44, 45). Indeed, T_RM_ expression and function are strictly dependent on local IL-15 levels (41), as shown by their absence in IL-15^–^/^–^ mice (43). On the other hand, in benign nevi, epidermis integrity is preserved, whereas the well-delimited foci of IL-15^+^ melanocytes appear in the dermis. In pathologic conditions, in cutaneous melanomas, IL-15^+^ tumor cells displayed a broad distribution that intensifies at different stages, becoming almost total in metastatic samples.

Subsequently, we investigated several primary and metastatic cell lines for the spontaneous or induced expression of IL-15Rα transcripts specifically involved in the assembly of membrane (WT IL-15Rα) or soluble (IC3 IL-15Rα) isoforms of the IL-15/IL-15Rα complex using PCR (12). Although most of the cell lines were expressed spontaneously or after IFN-α/anti CD40 stimulation with the above mentioned IL-15Rα transcripts, none of them released detectable amounts of the soluble complex. On the other hand, the mbIL-15 isoform which is likely to represent the predominant IL-15 isoform in melanomas was detected in all of them. Thus, the mbIL-15 isoform could act similarly to the soluble complex, anergizing infiltrated NK cells through long-term cell-to-cell contact mechanisms. Alternatively, an inflammatory microenvironment could favor the production of disintegrin and metalloproteinase (ADAM), which is effective in inducing the shedding of mbIL-15 (31), thus promoting its diffusion in the TME and exerting the immunoregulatory effects that we had initially attributed to juxtacrine IL-15 loops (10, 21). Concerning this point, our data show that the IL-15 that presents at the surface of melanoma cells can be partially cleaved by treatment with PMA, a powerful activator of ADAM17 (33), in the metastatic samples, but not in the primary cells. This evidence suggests the co-existence of two isoforms of IL-15 at the surface of metastatic samples: the former is sensitive to enzymatic cleavage (mb-IL-15) and the latter is resistant to ADAM17 (the transmembrane IL-15). Since it has been recently shown that the activation of ADAM17 by IL-15 limits human NK cell proliferation (32), we propose that cleavage of the mbIL-15 by ADAM17 causes the spread of a soluble biological active complex within the melanoma TME. In addition, since PMA may induce the increased transcription of ADAM17 through NFkB and ERK1/2 signaling (46), which can be activated by IL-15 (47), we speculate that, in metastatic melanomas, the cleaved biological active complex increasing the enzymatic activity of ADAM17, and its transcription creates a self-perpetuating deleterious loop, leading to the decreased proliferation of infiltrating NK cells (32). Since ADAM17 is constitutively expressed by the majority of the cellular components of the TME (33), cleaved mbIL-15 activates enzymatic activity and the transcription of ADAM17 on a very broad cellular spectrum that amplifies the IL-15/ADAM17 loop, thus generating an important immune escape mechanism in the metastatic tumors. Subsequently, we investigated the correlation between increased intra-tumoral IL-15 expression in stage IV with the plasmatic levels of the sIL-15/IL-15R complex in non-lympho-depleted metastatic melanoma patients. Our data show that 26% of these patients presented with consistently high plasmatic levels of this soluble complex at different time points, suggesting that secretion of the sIL-15/IL-15Rα complex may occur for a long period of time. Our data on metastatic melanoma patients differ from previously published data reporting high plasmatic levels of the sIL-15/IL-15Rα complex in all eight metastatic melanoma patients analyzed (23). However, a major difference between the two groups of metastatic patients is that those reported on by Bergamaschi et al. were lympho-depleted (23), a condition that causes the temporary suppression of “cellular sinks”, resulting in rapid increases in levels of homeostatic cytokines such as plasmatic IL-15 (48, 49). Relatedly, we show that after refeeding peripheral blood NK cells expanded for 12 days in rIL-2 with the engineered IL-15clpx (16), there was a significant decrease of NK growth rate and levels of cytotoxic activity against K562 and NALM-18 target cells.

Notably, although the *in vitro* exposure of hyperagonist freshly isolated NK cells for 5 days to IL-15 efficiently induced high levels of cytolytic activity against target cells, the addition of IL-15 hyperagonist to rIL-2 pre-activated NK cells resulted in a significant reduction of their cytolytic activity. This shows that *in vitro* exposure to IL-15 hyperagonist can indeed cause the direct functional impairment of pre-activated NK cells in the absence of autologous “regulatory” immune cells (26). In this context, previous studies have shown that melanoma cell lines can block NK cells’ lytic function through different mechanisms (30, 50, 51).

As a further step, we tried to corroborate the experimental data by associating a public dataset analysis on melanoma patients. This approach indicates that (i) in stages II and III, high IL-15, and IL-15Rα expression correlates with a good OS, but this positive correlation is lost in stage IV, and (ii) strong IL-15 and IL-15Rα expressions significantly correlate with the high level of expression of CD5, a marker present on infiltrating T lymphocytes (TILs), and of NKp46, a NK-specific activating receptor, and their high level of expression strongly correlates with a better OS in stages II and III, but not in stage IV. However, this last conclusion should be tempered and interpreted with caution due to the small number of patients listed in stage IV.

These data suggest that intra-tumoral IL-15 displays a biphasic role in melanoma evolution; there is a positive immune effect that is preserved up to stage III, but that is lost during stage IV. These events strongly correlate with the evolution in the interactions between intra-tumoral IL-15 and T cells reported in the recent literature (42, 43, 45). Indeed, T_RM_ cells (CD8^+^/CD103^+^/CD69^+^/CD44^+^) in normal skin lack immune checkpoints and are critical for protection against melanoma by eliminating major histocompatibility complex (MHC) class I positive melanoma cells (42–45). T_RM_ cells, generated in metastatic melanomas, are functional despite the expression of the immune checkpoints PD-1 and LAG3 (52). However, the excessive expression of immune checkpoints on T cells limits their cytotoxic potential. Indeed, scRNA-seq data from 25 melanoma patients identifies a large population of CD8^+^ T cells showing continuous progression from early effector CD8^+^ T cells into dysfunctional CD8^+^ T cells, characterized by the progressive activation of immune checkpoint gene expression (53). Thus, the IL-15-dependent proliferation of CD8^+^ T cells with high levels of expression of IL-15-induced immune checkpoints (54), associated with increased intra-tumoral IL-15 expression during stage IV, finally leads to the expansion of dysfunctional non-cytotoxic CD8^+^ T cells.

Human melanoma cells are characterized by the heterogeneous expression of HLA-I molecules. Thus, HLA-I negative melanoma cells will be unaffected by the activity of effector T cells (55), but susceptible to that of NK cells (35). In this context, repeated stimulation with IL-15 hyperagonists in pre-clinical models is characterized, during the first stimulation, by the synchronous amplification of NK cells and CD8^+^/CD44^+^ T cells. These T cells, upon successive rounds of stimulation, inhibit IL-15-induced NK cell expansion and their functional activation (26). Moreover, a recent study shows that a second cycle of the superagonist N-803 injection may lead to reduced NK cell response in patients bearing metastatic non-small cell lung cancer who were included in a phase 1b trial (56).

On the other hand, data from the literature show that in NK cells, the expression of immune checkpoints causes functional anergy, but with mechanisms different from those reported in T cells. For instance, resting and activated NK cells intracellularly express PD-1 RNA and protein that does not interfere with NK cytolytic function (57). In contrast, PD1 surface expression may be detected and mediate NK cell inhibition only in NK cells derived from neoplastic or CMV-infected patients (58, 59). Remarkably, PD-1 is detected on the surfaces of NK cells only in response to combined treatment with IL-12, IL-15, IL-18, and steroids (60). Moreover, a recent study demonstrated that LAG3 is expressed at very low levels in activated NK cells, but that its expression is substantially increased in adaptive NK cells that are chronically exposed to CMV-infected cells (61). Importantly, these cells demonstrate low activity against infected tumor cells. Thus, based on these results, we propose that during stages II and III, hyperagonist IL-15 isoforms, present in the TME, may stimulate NK cell cytotoxicity. In contrast, during stage IV, the increased levels of hyperagonist IL-15, sustained over a long period, and of additional soluble inhibitory factors (59) in the TME could cause NK cell functional impairment also through the induced expression of PD-1 and LAG3 (58–61).

Thus, continuous stimulation with IL-15 hyperagonist over time could lead to NK cells becoming hypo-responsive and therefore incapable of killing HLA-I low/negative melanoma cells (62), and also to dysfunctional T_RM_-like cells being unable to eliminate HLA-I positive melanoma cells (31, 53). Therefore, based on the above mentioned data, we propose that an imbalance in the production/secretion of the sIL-15/IL-15Rα complex may occur in a subgroup of metastatic patients. This could lead to permanently high plasmatic levels of the soluble complex that could directly or indirectly impair the function of autologous NK cells or, in adoptive immunotherapy, of infused pre-activated NK cells, pointing to the existence of a novel NK cell immune escape mechanism.

In conclusion, melanoma appears to display complex mechanisms of immune activation and regulation that have not been completely clarified, and in which, for instance, the possible role of IL-15/IL-15Rα complex and isoforms has been underestimated (60). In this context, several reports indicate a constant rise in the incidence of melanoma in childhood and adolescence, and this trend is largely due to increased UV exposure (1). Since skin exposure to UV-B rays causes rapidly increased production of IL-15 (6), which modifies the cutaneous microenvironment since childhood, it seems conceivable that this event may be related both to the early development of pediatric melanomas and to the appearance of delayed lesions in adults. Thus, we propose that the presence of mbIL-15 and secreted IL-15/IL-15Rα complexes could in early tumor stages promote the production of cytotoxic lymphocytes cells (T and NK cells), but promote the accumulation of dysfunctional tumor infiltrated lymphocytes in stage IV. It is possible that this bias could be counteracted by therapeutic strategies that combine the use of checkpoint inhibitors and IL-15 inhibitors, such as neutralizing IL-15 monoclonal antibodies (mAbs) or small chemical compounds (63).

## Data availability statement

The original contributions presented in the study are included in the article/**Supplementary Material**. Further inquiries can be directed to the corresponding author.

## Ethics statement

The use of human overflow plasmas and the research protocol were approved by the Clinical and Research Center and the National Cancer Institute (IST, Genoa, Italy -OMA12.007- and by “comitato etico regionale CER Liguria”: 046REG2017. Buffy coats were collected from volunteer blood donors admitted to the blood transfusion service of OPBG. The Ethical Committee of OPBG approved the study (825/2014) that was conducted in accordance with the ethical principles stated in the Declaration of Helsinki.

## Author contributions

Contributed to research conception and planning: BA, IC, LM, EMo, CS, EMa, and SDM. Development of methodology: SDM, PFF, AP, NT, VP, FRM, SS, CS, SE, MM, and DH. Performed experiments, acquisition of data, acquired and managed patients, provided facilities, and so on: SDM, PFF, NT, SS and CS. Analysis and interpretation of data: BA, CI, PV, and SDM. Performed immunohistochemical analysis: MP, EMu and SDM. Performed dataset analysis: SE, MM, DH, and SDM. Study supervision: BA, IC, LM, EMa, EMo, PV, and SDM. Writing, review, and/or revision of the manuscript: BA, IC, LM, PV, NT, EMa, EMo, and SDM. Yielded materials essential for the research and contributed to project planning: GP, MG, EMo, SB. All authors contributed to the article and approved the submitted version.

## Supplementary material

Supplementary Figure 1 Correlation between IL-15, IL-15Rα, CD5 and NKp46. Correlation between CD5, NKp46, and OS at different stages. Using the public dataset (TCGA), different analyses were performed. Correlation between expression of IL-15, IL-15Rα, CD5 (T cell marker), and NKp46 (NK marker) in human melanomas **(A)**. Panels **(B, C)** show the OS based on the levels of expression of CD5 and NKp46 at the different stages of melanomas. IL-15, interleukin-15; NK, natural killer; OS, overall survival; TCGA, The Cancer Genome Atlas.
