# Supplementary figures and images for: The roles of different forms of IL-15 in human melanoma progression
Source: Front Immunol. 2023 Jun 2;14:1183668. doi: 10.3389/fimmu.2023.1183668 (PMC10272795; doi:10.3389/fimmu.2023.1183668)

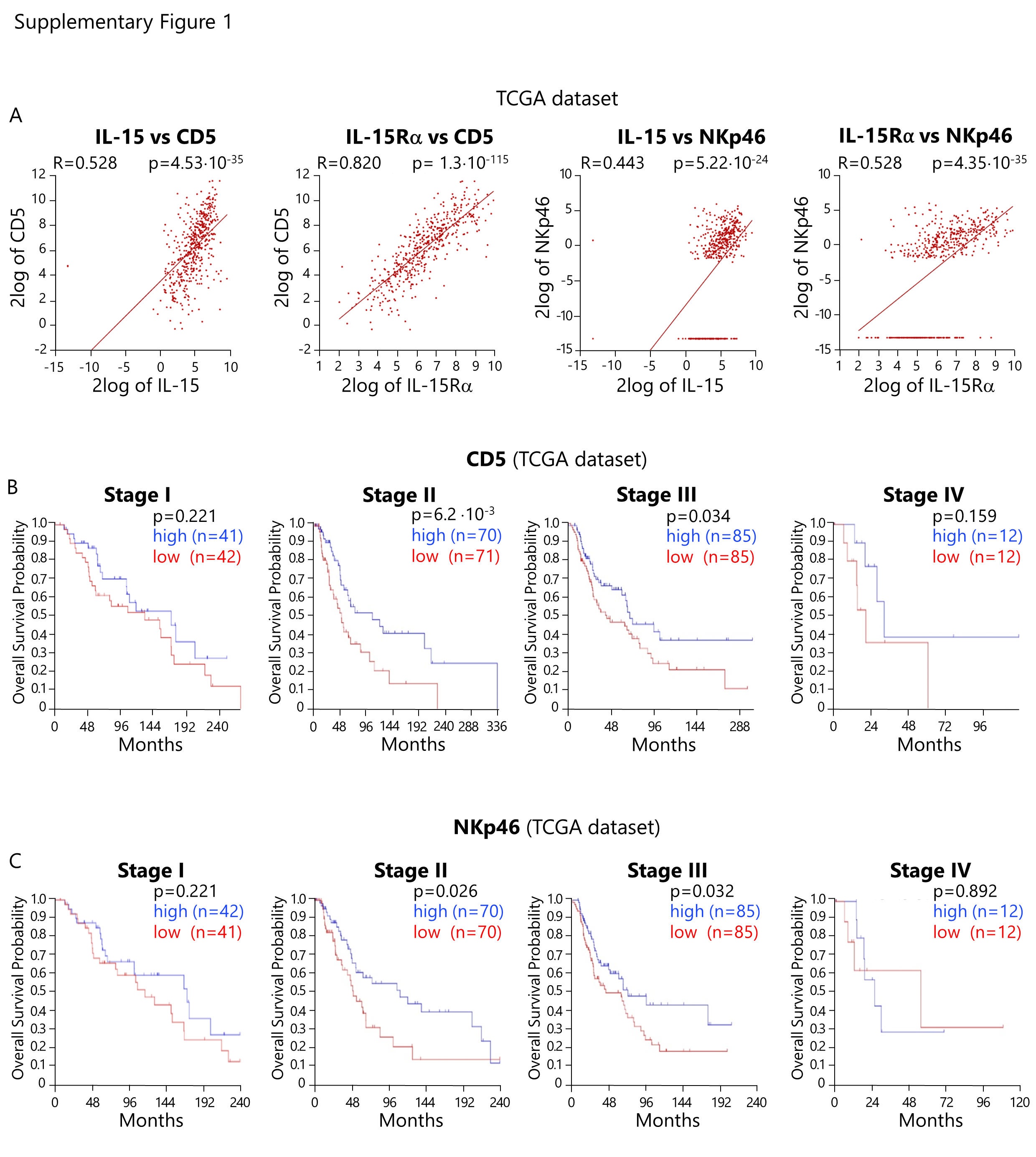

Supplement: Supplementary Figure 1 — Correlation between IL-15, IL-15Rα, CD5 and NKp46. Correlation between CD5, NKp46, and OS at different stages. Using the public dataset (TCGA), different analyses were performed. Correlation between expression of IL-15, IL-15Rα, CD5 (T cell marker), and NKp46 (NK marker) in human melanomas (A). Panels (B, C) show the OS based on the levels of expression of CD5 and NKp46 at the different stages of melanomas. IL-15, interleukin-15; NK, natural killer; OS, overall survival; TCGA, The Cancer Genome Atlas. [file Image_1.tif]
